# Improved cell metabolism prolongs photoreceptor survival upon retinal-pigmented epithelium loss in the sodium iodate induced model of geographic atrophy

**DOI:** 10.18632/oncotarget.7330

**Published:** 2016-02-11

**Authors:** Marina Zieger, Claudio Punzo

**Affiliations:** ^1^ Department of Ophthalmology and Gene Therapy Center, University of Massachusetts Medical School, Worcester MA, USA

**Keywords:** AMD, cone degeneration, rod degeneration, geographic atrophy, mTORC1, Gerotarget

## Abstract

Age-related macular degeneration (AMD) is characterized by malfunction and loss of retinal-pigmented epithelium (RPE) cells. Because the RPE transfers nutrients from the choriocapillaris to photoreceptor (PR), PRs are affected as well. Geographic atrophy (GA) is an advanced form of AMD characterized by severe vision impairment due to RPE loss over large areas. Currently there is no treatment to delay the degeneration of nutrient deprived PRs once RPE cells die. Here we show that cell-autonomous activation of the key regulator of cell metabolism, the kinase mammalian target of rapamycin complex 1 (mTORC1), delays PR death in the sodium iodate induced model of RPE atrophy. Consistent with this finding loss of mTORC1 in cones accelerates cone death as cones fail to balance demand with supply. Interestingly, promoting rod survival does not promote cone survival in this model of RPE atrophy as both, rods and cones suffer from a sick and dying RPE. The findings suggest that activation of metabolic genes downstream of mTORC1 can serve as a strategy to prolong PR survival when RPE cells malfunction or die.

## INTRODUCTION

Age-related macular degeneration (AMD) is one of the leading causes for visual impairment in the industrialized world [1]. The prevalence increases with age affecting one in four people by age 80 [2]. AMD primarily causes photoreceptor (PR) loss in the central area of the retina that is specialized for high-acuity color vision. The initial pathogenesis of AMD is characterized by the formation of drusen, which are deposits of cellular debris that accumulate on the basal side of the retinal-pigmented epithelium (RPE), followed by abnormalities in retinal pigmentation, the Bruch's membrane, and loss of choriocapillaries [3-7]. Severe vision impairment occurs as the disease progresses to an advanced stage where large areas of RPE are lost. This stage, which is commonly referred to as geographic atrophy (GA), directly affects PRs as the RPE transfers nutrients from the choriocapillaries to PRs [8, 9]. Currently there is no therapy to treat this advanced stage of AMD that is characterized by RPE atrophy [9, 10], although a number of embryonic stem cell based RPE therapies are being evaluated to replace sick or lost RPE cells [11-14].

Most studies on AMD center on deciphering the disease progression of the initial stages, meaning understanding the order in which the mutualistic symbiotic relationship between the RPE, the underlying Bruch's membrane and the choriocapillaris is lost [15]. However, regardless of the sequence of events, collapse of the RPE/Bruch's membrane/choriocapillaris complex in advanced stages of AMD directly affects PR homeostasis due to reduced nutrient availability. Here we attempt to test if it is possible to directly prolong PR survival when the RPE dies. To that end we used the sodium iodate induced mouse model of acute RPE atrophy and tested a strategy that we have successfully employed to prolong the survival of nutrient deprived cones in retinitis pigmentosa [16, 17]. By promoting cell metabolism through activation of the kinase mammalian target of rapamycin complex 1 (mTORC1), which balances demand with supply [18], secondary cone death can be significantly delayed in retinitis pigmentosa [16]. We therefore genetically activated the pathway in cones, and in a separate experiment in rods, of mice injected with sodium iodate and tested the role of pro-growth mechanisms that are controlled by mTORC1 and pro-survival mechanisms that are controlled by mTORC2 (Supplemental Figure 1). Sodium iodate is a strong oxidizing agent that preferentially affects RPE cells causing rapid RPE atrophy followed by PR loss, mimicking changes seen in patients with GA [10, 19-22]. We found that constitutive activation of mTORC1 in cones was sufficient to promote cone survival after sodium iodate injection while loss of mTORC1 activity accelerated cone loss. In contrast, loss of mTORC2 activity in cones did not affect cone survival in animals injected with sodium iodate. Similar to the findings in cones, constitutively activated mTORC1 in rods promoted rod survival. However, improved cell-autonomous rod survival did not promote cone survival as both PR cell types suffered from the loss of the overlying RPE. Improved PR survival was accompanied by increased expression in key metabolic enzymes. The data shows that promoting pro-growth mechanisms in PRs prolongs PR survival after acute RPE loss suggesting that nutrient deprivation may be the major cause for the demise of PRs in AMD. Additionally, in conjunction with our previous analysis on the role of mTORC1 in retinitis pigmentosa [16] the data suggests that even though the pathologies and the origins of retinitis pigmentosa and AMD are quite different, both PR degenerative diseases may be treated with the same therapeutic approach to prolong PR survival.

## RESULTS

### Calibrating the sodium iodate model of GA

**Figure 1:**
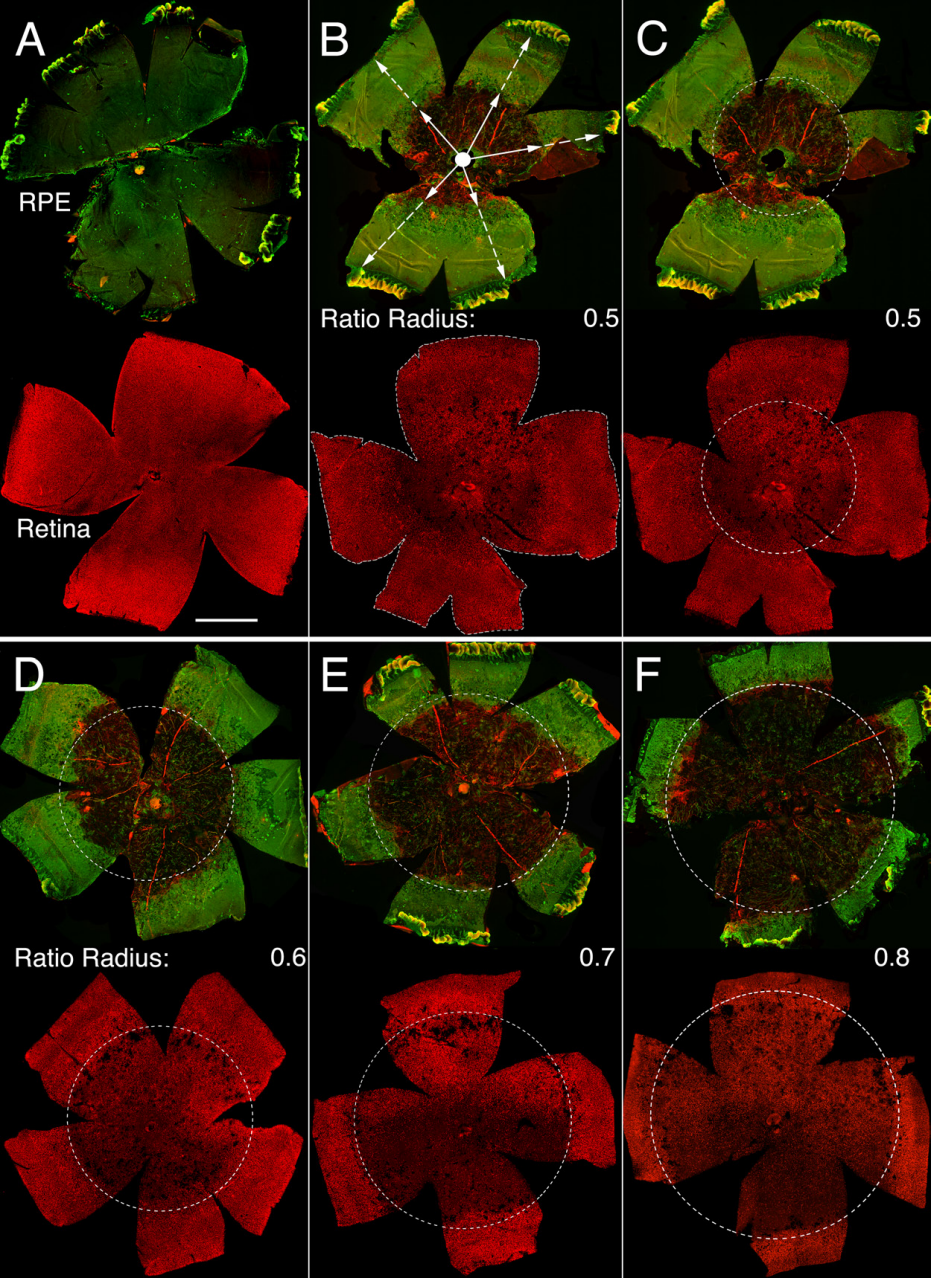
Sodium iodate induced RPE and retinal atrophy **A.**-**F.** Representative RPE (upper rows) and retinal flat mounts (lower rows) from same eye of an uninjected control animal (A) and animals injected with sodium iodate (B-F) and analyzed 4 weeks post-injection. **B.** Representative RPE flat mount (upper panel) with an average damage radius of 0.5. The average damage radius is calculated by averaging the ratios obtained from dividing the length of the short radius (full arrow) by the length of the full radius (dotted arrow). (B: lower panel) Retinal flat mount corresponding to RPE flat mount in top panel highlighting the entire retinal surface area used to calculate cone survival by determining the amount of colocalization of the red signal (cone arrestin) over the entire retinal surface area (dotted line). **C.** Same panels as in (B) indicating the area of RPE damage (dotted circle) with a damage radius of 0.5 and the corresponding area on the retinal flat mount (dotted circle) in which cone survival was assessed by determining the percentage of the red signal (cone arrestin) in the circled area. **D.**-**F.** Similar examples as in (C) with increasing radius of RPE damage. (Upper row in each panel: green signal shows GLUT1 expression while red signal shows F-actin identified by rhodamine phalloidin, because the RPE is not damaged in (A) GLUT1 signal is predominant in the control; lower row in each panel: red signal shows cone arrestin). Scale bar: 1 mm.

Systemic injection of sodium iodate has been widely used to study AMD and GA because it preferentially affects RPE cell health, function and survival, followed by loss of PRs [10]. However, because this model depends on the targeting efficiency of the tail vein injection (Supplemental Table 1), there can be quite a large variability in the effects on the RPE. To ensure that differences in photoreceptor survival were not due to differences in RPE loss, RPE damage was evaluated in each animal on flat mounts before including the animal into the PR survival study. The radius of concentric RPE atrophy, which expanded from the optic nerve head outwards, was measured and divided by the radial distance from the optic nerve head to the *ora serrata* (Figure 1). This ratio, which ranges from 0 to 1, was reflective of the extent of the major radial RPE damage (hence on referred to as RPE damage radius). The major area of RPE damage was readily visible by immunofluorescence due to the absence of glucose transporter 1 (GLUT1) expression, normally found on the apical and basolateral RPE plasma membrane [23, 24]. In addition, loss of RPE cells made the underlying choriocapillary network that was also visualized by GLUT1 and rhodamine labeled phalloidin visible, allowing for a second indepedent demarcation of the RPE area that was lost (Figure 1). Photoreceptor survival was scored only on retinas with an RPE damage radius of ≥0.5. No RPE damage radius exceeded 0.8. Because the severity of PR degeneration followed the extent of RPE degeneration, the presence of cones was scored over the entire retinal surface area, as well as over a concentric area corresponding to the area of RPE damage of the same eye (Figure 1). Rod degeneration was scored on retinal cross-sections, where it became apparent that there were 3 zones of degeneration with the far periphery being unaffected (Supplemental Figure 2). Therefore, outer nuclear layer (ONL) thickness was not recorded in the last 20% of the cross-sections.

### Activation of the insulin/mTOR pathway in cones

**Figure 2:**
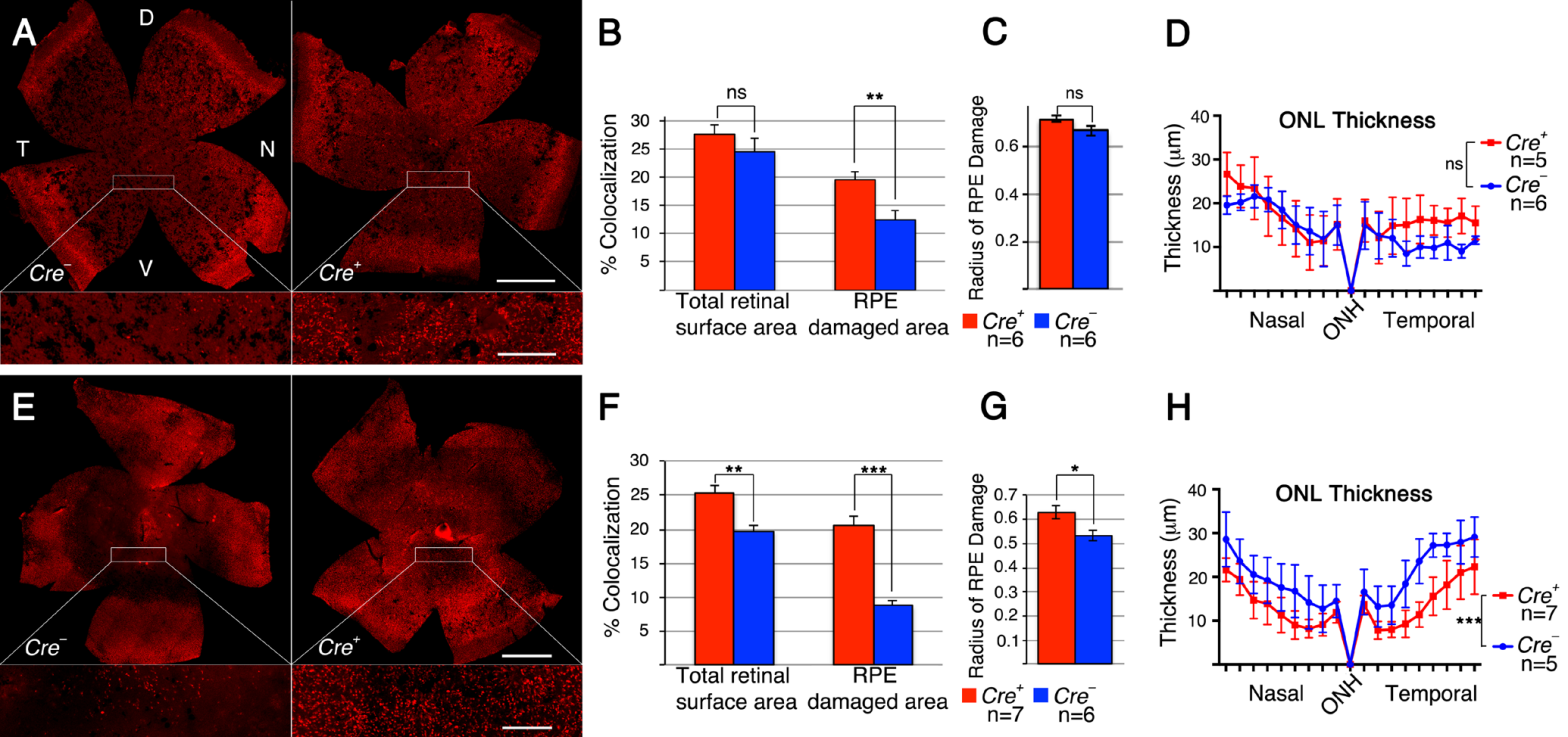
Loss of *Pten* or *Tsc1* in cones prolongs cone survival **A.**-**H.** Analyses of M*Cre+* and M*Cre-* littermates harboring the *Ptenfl/fl* alleles (A-D) and the *Tsc1fl/fl* alleles (E-H) at 4 weeks post sodium iodate injection. **A.**, **E.** Representative retinal flat-mounts showing higher cone density in M*Cre+* animals (see higher magnification in boxed area; red signal: cone arrestin). Dorsal (D), temporal (T), ventral (V) and nasal (N) orientations are indicated. Orientation in (E) is the same as in (A). **B.**, **F.** Percentage colocalization between cone arrestin and surface area indicated. Upon loss of *Pten* only the surface area corresponding to the RPE damage shows a statistically significant difference, while upon loss of *Tsc1* there is also a statistically significant differences when the colocalization is calculated over the entire retinal surface area. **C.**, **G.** Average of RPE damage radius and ONL thickness diagram **D.**, **H.** depicting rod survival. There was more RPE damage in M*Cre+* animals of the *Tsc1* conditional allele (G), which is reflected in the thickness of the ONL (H). Scale bars in (A, E): 1mm (upper) and 200 μm (lower). (n: number of animals analyzed; ns: not significant; *: *p* < 0.05); **: *p* < 0.01; ***: *p* < 0.005).

To test if improved cell metabolism by sustained activation of the insulin/mTOR pathway prolongs cone survival in the sodium iodate induced model of RPE atrophy, we constitutively activated the pathway in cones at two separate junction points downstream of the insulin receptor (Supplemental Figure 1). This was achieved by use of the *Cre*-lox system to conditionally delete the tumor-suppressor gene phosphatase and tensin homolog (*Pten*) [25], and separately the tuberous sclerosis complex 1 (TSC1) [26] gene *Hamartin* (hereafter referred to as *Pten*cKO or *Tsc1*cKO: cKO = cone knockout; in all instances M*Cre+* denotes cKO of conditional allele indicated; M*Cre+*: cone specific Cre-recombinase line [27]). *Pten* is a phosphatase that decreases the intracellular second messenger levels of phosphatidylinositol-trisphosphate (PIP_3_) thereby balancing growth factor signals. Because PIP_3_ levels remain high in the absence of *Pten,* the pathway is constitutively activated resulting in increased activity of the pro-growth kinase mTORC1, and the pro-survival kinases mTORC2 and AKT (Supplemental Figure 1). In contrast to loss of *Pten*, which activates all components downstream of the insulin receptor, loss of *Tsc1* activates only mTORC1 allowing us to differentiate between pro-growth and pro-survival mechanisms. We found that sustained pathway activity in cones by loss of either *Pten* or *Tsc1* (Figure 2A, 2B, 2E, 2F) significantly improved cone survival at 4 weeks post-sodium iodate injection. However, after the loss of *Tsc1*, we measured a statistically significant difference in cone survival between M*Cre-* and M*Cre+* animals over the entire retinal surface area and the surface area corresponding to the area of RPE damage (Figure 2F). In contrast, after the loss of *Pten*, cone survival was only improved in the area where the RPE was damaged (Figure 2B). This suggests that loss of *Pten* was less efficient in delaying cone death when compared to loss of *Tsc1*. Further evidence for this finding is provided by the fact that RPE damage was similar between M*Cre-* and M*Cre+* animals upon loss of *Pten* (Figure 2C) and statistically higher in M*Cre+* animals upon loss of *Tsc1* (Figure 2G), nonetheless, loss of *Tsc1* still improved cone survival even when measured over the entire retinal surface area. To test whether the difference in cone survival between loss of *Pten* and *Tsc1* correlated with strength in pathway activation, we analyzed the expression of phosphorylated ribosomal protein S6 (p-S6), a *bona fide* downstream target of mTORC1. Immunofluorescence analyses on retinal cross sections of uninjected animals revealed that p-S6 levels were much higher in cones that lacked *Tsc1* (Supplemental Figure 3A-3C), indicating that higher mTORC1 activity was likely responsible for the difference seen in cone survival upon loss of *Pten* and *Tsc1*.

Evaluation of rod survival across the nasal-temporal axis showed a dramatic reduction in ONL thickness (Figure 2D, 2H) when compared to uninjected control animals (Supplemental Figure 4). In animals harboring the *Pten* conditional allele there was no statistically significant difference in ONL thickness between M*Cre-* and M*Cre+* mice (Figure 2D). These results indicate that cone survival was compared between two groups of animals with a similar extend of retinal (Figure 2D) and RPE damage (Figure 2C). In contrast, in mice harboring the *Tsc1* conditional allele there was a statistically significant difference in ONL thickness (Figure 2H). M*Cre+* animals suffered clearly more damage paralleling the analysis of the RPE damage radius (Figure 2G). However, in spite of the higher number of surviving rods in M*Cre*- animals (Figure 2H) the number of surviving cones was significantly higher in M*Cre+* retinas suggesting that loss of *Tsc1* efficiently promotes cone survival.

The toxic insult of sodium iodate, which led to rapid RPE degeneration, also dramatically affected retinal physiology. Electroretinogram (ERG) recordings at 2 weeks post-injection showed a rapid and statistically significant drop in the PR a-wave and inner nuclear layer (INL) cells b-waves of the scotopic rod and photopic cone responses (Supplemental Figure 5). This initial decline was so steep that no further decline was recorded by 4 weeks post-injection with the exception of the scotopic a-wave (Supplemental Figure 5). Consequently, no statistically significant difference in the photopic response was seen between M*Cre-* and M*Cre+* animals (Supplemental Figure 5) when either *Pten* or *Tsc1* was deleted in cones despite improved cone survival. Interestingly, because the a-wave is generated by PRs and continued to decline in case of the rods between 2 and 4 weeks as the more peripheral rods died, while the b-wave, which is generated mainly by bipolar cells, reached its low point by 2 weeks, the data suggests that INL cells are affected as well by sodium iodate. This finding is consistent with data from others, showing that sodium iodate causes loss of the photoreceptors synaptic marker bassoon [21, 28]. Despite these circumstances, the drop in the b-wave amplitudes for photopic and scotopic response at 2 weeks of age (Supplemental Figures 5, 6) were reliable indicators that the sodium iodate injection was successful and the RPE damage radius would be at least 0.5.

**Figure 3:**
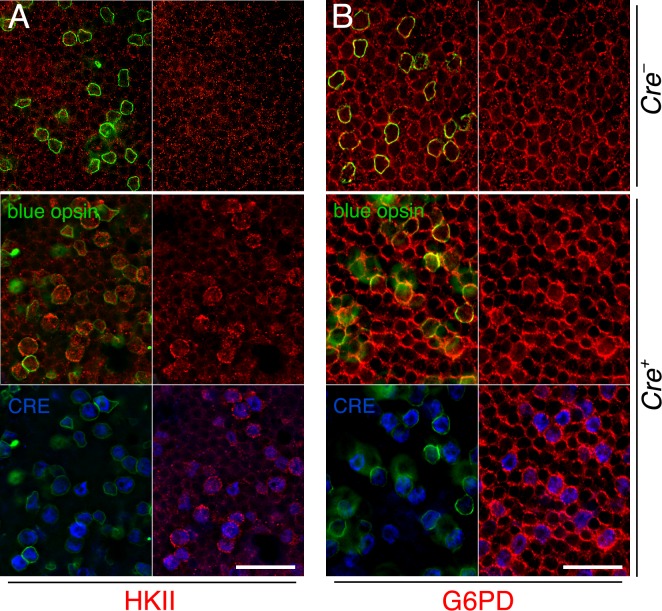
Increased expression of metabolic enzymes in cones upon activation of mTORC1 **A.**, **B.** Immunofluorescence analyses on retinal flat mounts of M*Cre-* (first row) and M*Cre+* (rows 2 and 3) littermates harboring the *Tsc1fl/fl* alleles at 4 weeks post sodium iodate injection. Increased immunofluorescence signal for HKII (A: red signal) and G6PD (B: red signal) is see in CRE (blue signal) positive cones identified also by the expression of short wave length opsin (blue opsin; green signal). In CRE negative retinas (first row) expression of HKII and G6PD appears uniform between the remaining cones (green signal) and rods. Scale bars: 25 μm.

mTORC1 is know to control the expression of a metabolic gene regulatory network including glycolysis, fatty acid synthesis, and the pentose phosphate pathway [18]. We previously found that improved cone survival due to elevated mTORC1 activity in cones of retinitis pigmentosa animals is accompanied by increased expression of metabolic genes [16]. To evaluate if the same mechanism was occurring after sodium iodate injection we analyzed the expression of hexokinase II (HKII) and glucose-6-phosphate dehydrogenase (G6PD) in M*Cre-* and M*Cre+* mice harboring the *Tsc1* conditional allele. We found increased expression of HKII and G6PD in M*Cre+* cones of injected animals (Figure 3) suggesting that the same mechanism of protection prolongs cone survival in two different retinal degenerative diseases.

In summary, the data show that activating the insulin/mTOR pathway in cones of mice with acute RPE loss promotes cone survival. While loss of either *Pten* or *Tsc1* delays cone death, loss of *Tsc1* appears more efficient in prolonging cone survival as it activates mTORC1 more robustly. This indicates that increasing mTORC1 activity alone is sufficient to promote cone survival when RPE cells die.

### Role of the two mTOR Complexes during cone death

**Figure 4:**
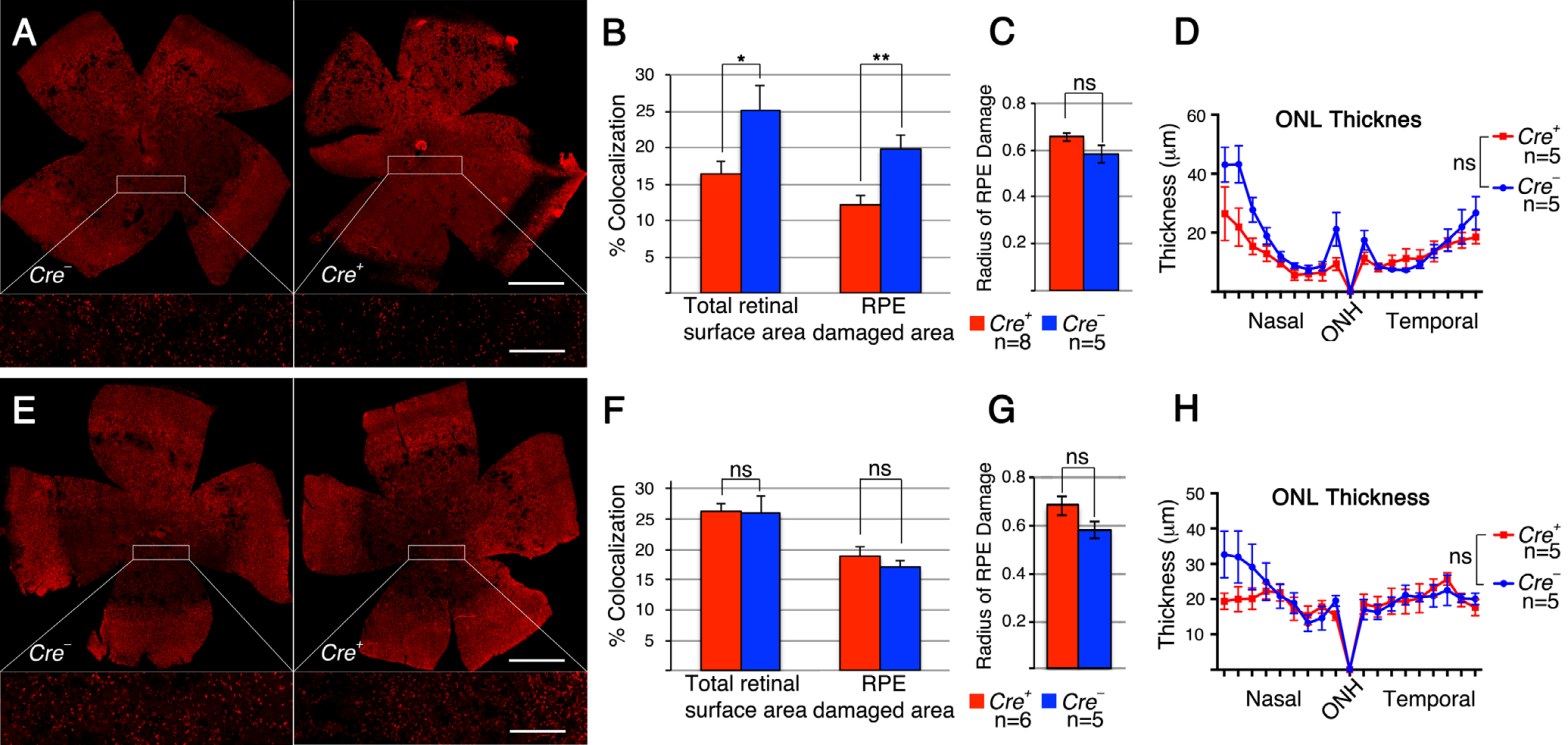
Loss of *Raptor* but not *Rictor* in cones accelerates cone death **A.**-**H.** Analyses of M*Cre+* and M*Cre-* littermates harboring the *Raptorfl/fl* alleles (A-D) and the *Rictorfl/fl* alleles (E-H) at 4 weeks post sodium iodate injection. **A**., **E**. Representative retinal flat-mounts showing lower cone density in M*Cre+* animals with the *Raptor* conditional allele. Orientation in (A, E) is the same as shown in Figure 2A. **B**., **F**. Percentage colocalization between cone arrestin and surface area indicated. Upon loss of *Raptor* there is a statistically significant drop in cones. **C**., **G**. Average of RPE damage radius. **D**., **H**. ONL thickness diagram depicting rod survival. There is no difference in RPE damage and ONL thickness between M*Cre+* and M*Cre-* animals. Scale bars in (A, E): 1mm (upper) and 200 μm (lower). (n: number of animals analyzed; ns: not significant; *: *p* < 0.05); **: *p* < 0.01).

Our data show that increasing mTORC1 activity is sufficient to promote cone survival when RPE cells die. However, the experiments did not address if the metabolic problems in cones are the main reason for cone death or if loss of RPE secreted growth factor support during disease contributes to PR death as well [29]. To further test the role of cell metabolism and growth factors, we investigated whether loss of mTORC1 activity and separately, loss of mTORC2 affects cone survival after sodium iodate injection. To that end we deleted their unique accessory proteins RAPTOR and RICTOR, respectively, using the same cone specific M*Cre* driver crossed to a *Raptor* and separately, a *Rictor* conditional allele. We found that loss of *Raptor* (mTORC1) dramatically accelerated cone death after sodium iodate injection, while loss of *Rictor* had no effect on cone survival (Figure 4A, 4B, 4E, 4F). In both cases there was no statistically significant difference in RPE damage between M*Cre+* and M*Cre*- animals (Figure 4C, 4G). Consistent with these results ONL thickness was similar within the same genetic background (Figure 4D, 4H), indicating that cone survival was compared between animals that suffered the same extent of RPE and rod cell loss. Similar to our previous observations with the *Pten* and *Tsc1* conditional allele, PR function declined in both cases rapidly (Supplemental Figures 5, 6). The data suggests that mTORC1 activity is required to help cones balance demand with supply when RPE cells die, implying a nutrient shortage in cones. It further suggests that loss of growth factor mediated signals that depend on mTORC2 during disease do not significantly contribute to cone survival when the RPE dies.

### Activation of mTORC1 in rods

**Figure 5:**
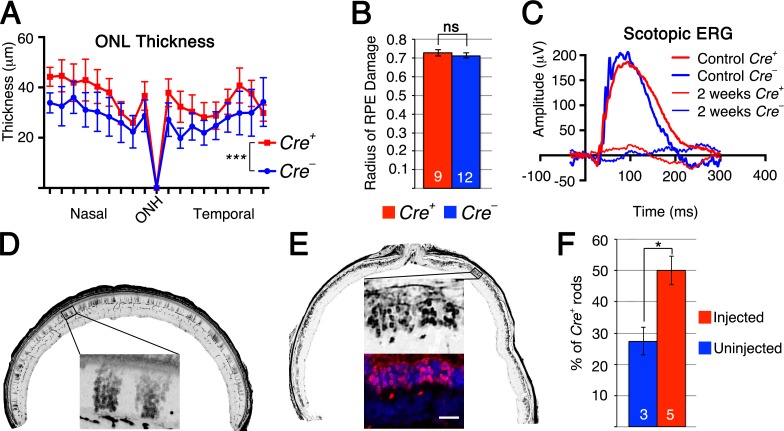
Loss of *Tsc1* in rods promotes rod survival **A.**-**F.** Analyses of R*Cre+* and R*Cre*- littermates harboring the *Tsc1fl/fl* alleles at 2 weeks post sodium iodate injection (D: uninjected). **A.** ONL thickness diagram showing quantification of rod survival. **B.** Average of RPE damage radius. **C.** Representative scotopic ERG recordings of uninjected control at 1 month of age and injected animals at 2 weeks post-injection. **D.**, **E.** Representative retinal sections along the nasal temporal axis used to count CRE+ rods showing patchy distribution of R*Cre+* rods in uninjected control mouse (D) and increased density of R*Cre+* rods in injected mouse (E) (red signal indicates *Cre* recombinase; blue signal indicates DAPI; other images are in gray scale). **F.** Percentage of R*Cre+* rods over total ONL cell number in uninjected control and injected experimental animals. (Numbers in bars indicate number of animals analyzed; *: *p* < 0.05; ***: *p* < 0.005). Scale bar: 25 μm (E).

It is generally understood form studies on retinitis pigmentosa that saving rods equates to saving cones. We therefore investigated if loss of *Tsc1* in rods would also promote rod survival after sodium iodate injection, and if so, would that affect cone survival. The same *Tsc1* conditional allele was crossed to a rod-specific Cre-driver line (R*Cre+*) [30] and retinas were analyzed at 2 weeks post sodium iodate injection as our previous experiments showed already a very large loss of rods by 4 weeks post-injection (Supplemental Figure 2). Measurements of the ONL thickness revealed a statistically significant greater number of surviving rods in R*Cre+* retinae when compared to R*Cre*- controls with a similar extent of sodium iodate induced RPE damage (Figure 5A, 5B) between R*Cre+* and R*Cre-* controls. Similar to our previous findings, rod cells function declined rapidly (Figure 5C and Supplemental Figure 5) showing no significant difference between R*Cre+* and R*Cre-* animals. While rod survival was clearly improved, the difference between R*Cre+* and R*Cre-* mice was only around 5-10μm at each intersection measured, corresponding to about 1-2 rows of rods. To investigate why a greater number of rods were not surviving we analyzed the levels of p-S6 in rods of uninjected animals. Surprisingly, p-S6 levels in rods were more comparable to p-S6 levels in cones in which *Pten* was deleted rather than to cones in which *Tsc1* was ablated (Supplemental Figure 3D). This suggests that mTORC1 is activated to a lesser extent in rods upon loss of *Tsc1* than in cones, possibly explaining why not more rods survive upon loss of *Tsc1*.

**Figure 6:**
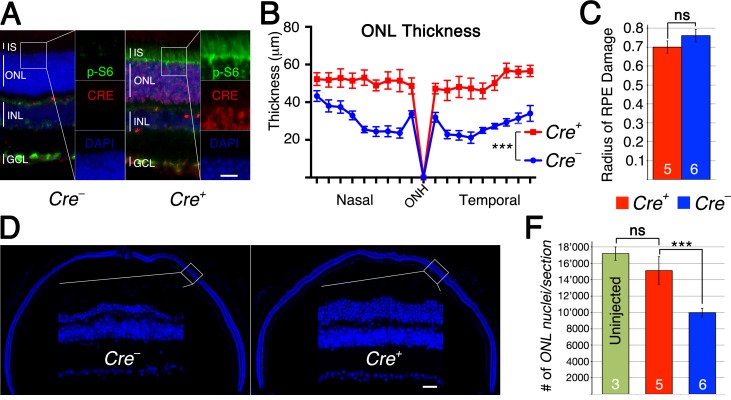
Loss of *Tsc1* in rods by i*Cre*75 **A.**-**F.** Analyses of i*Cre+*75 and i*Cre-*75 littermates harboring the *Tsc1fl/fl* alleles. **A.** Immunofluorescence analyses on retinal cross sections of uninjected animals showing increased p-S6 levels (green signal) in inner segments (IS) and ONL of *Cre+* animals. **B.** ONL thickness diagram showing quantification of rod survival. **C.** Average of RPE damage radius. **D.** Representative retinal sections along the nasal temporal axis used to count ONL nuclei (blue: nuclear DAPI). **F.** Quantification of ONL nuclei per section including uninjected control mice. Scale bar: 25 μm. Vertical lines in (A) mark thickness of IS, ONL, INL and gaglion cell layer (GCL). (Numbers in bars indicate number of animals analyzed; ***: *p* < 0.005).

The p-S6 staining in rods indicated that a smaller than expected number of rods express CRE recombinase. The original characterization of the rod *Cre* driver line used in this experiment reported Cre-recombinase expression in about 77% of rods (LMOPC1-Cre) [30]. A second line presented in the same publication with a different promoter reported expression of CRE in about 43% of all rods. We therefore decided to quantify the number of CRE+ rods in injected and uninjected animals lacking *Tsc1* in rods (Figure 5D, 5E) reasoning that if CRE+ rods have no survival advantage the overall percentage of CRE+ rods should remain the same after sodium iodate injection. Consistent with our ONL measurements (Figure 5A), we found that the overall percentage of CRE+ rods climbed from 28% in uninjected animals to 50% in sodium iodate injected animals by 2 weeks post injection indicating that CRE+ rods have a survival advantage (Figure 5F). To test if the difference in mTORC1 activation seen between rods and cones upon loss of *Tsc1* (Supplemental Figure 3D) affected the efficiency in rod survival we used a second rod-specific *Cre* line referred to as the i*Cre*75 line [31]. This *Cre* line expresses CRE recombinase in all rods (Figure 6A) and should result in more efficient rod survival if activation levels of mTORC1 in rods are less critical as in cones, where loss of *Pten* resulted in less efficient delay of cone death when compared to loss if *Tsc1*. Consistent with the presence of CRE in all rods the difference in ONL thickness (Figure 6B) between *Cre+* and *Cre-* littermates increased almost by 3 fold when compared to the R*Cre* line. Counting of the ONL nuclei (Figure 6D, 6F) revealed that 88% of ONL nuclei were preserved in *Cre+* animals at 2 weeks post-injection compared to 57% in *Cre-* animals. The experiments show that rods can also be rendered more resistant to the loss of RPE cells by activation of mTORC1 and that less robust activation of mTORC1 in rods when compared to cones is still sufficient to efficiently promote rod survival.

**Figure 7:**
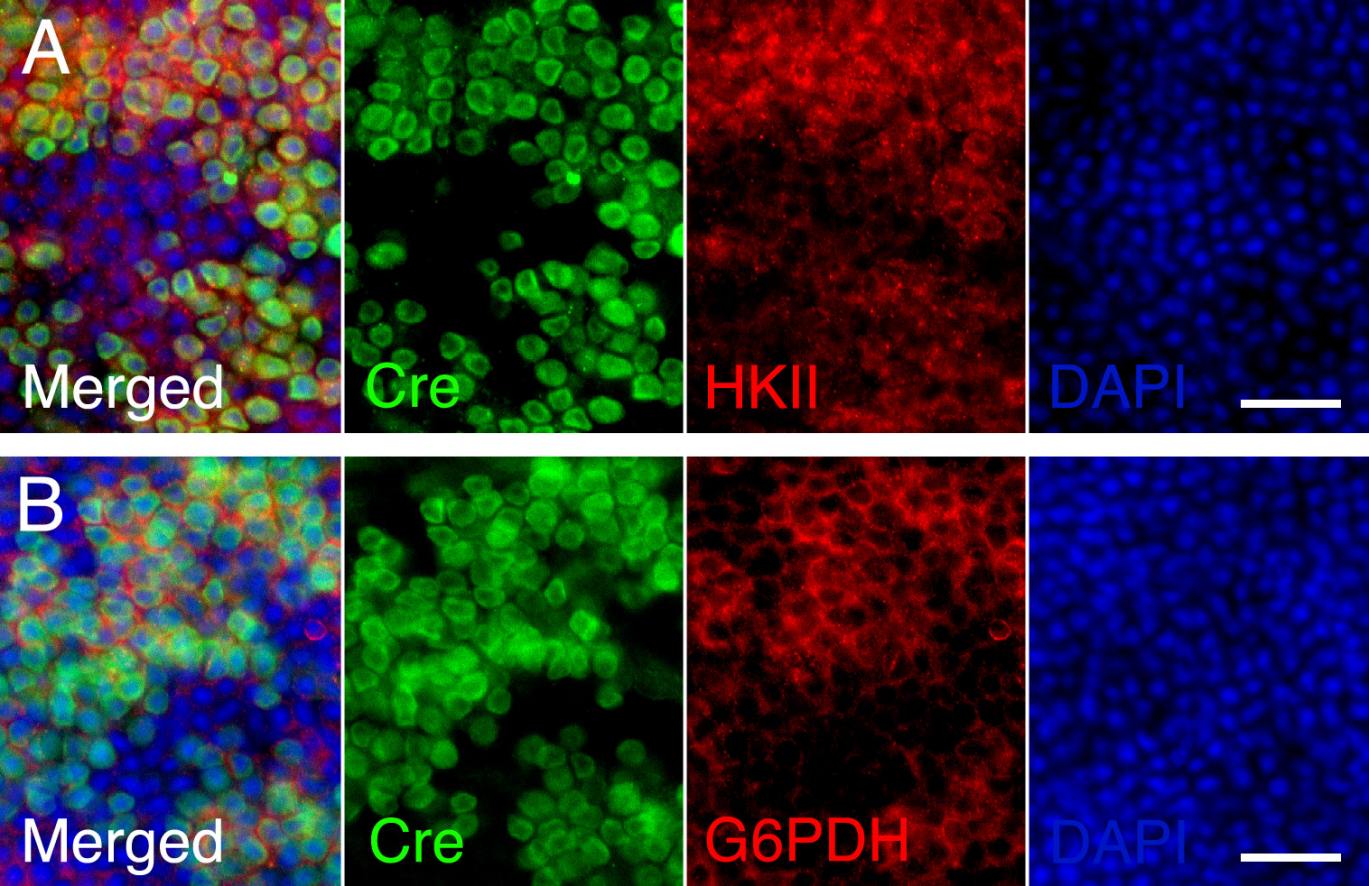
Increased expression of metabolic enzymes in CRE positive rods upon activation of mTORC1 **A.**-**B.** Immunofluorescence analyses on retinal flat mounts of R*Cre+* mice harboring the *Tsc1fl/fl* alleles at 2 weeks post-injection. Increased immunofluorescence signal for HKII (A: red signal) and G6PD (B: red signal) is seen in CRE positive rods while CRE negative rods and cones display less immunofluorescence. Scale bars: 25 μm. (Green: CRE; blue: nuclear DAPI).

**Figure 8:**
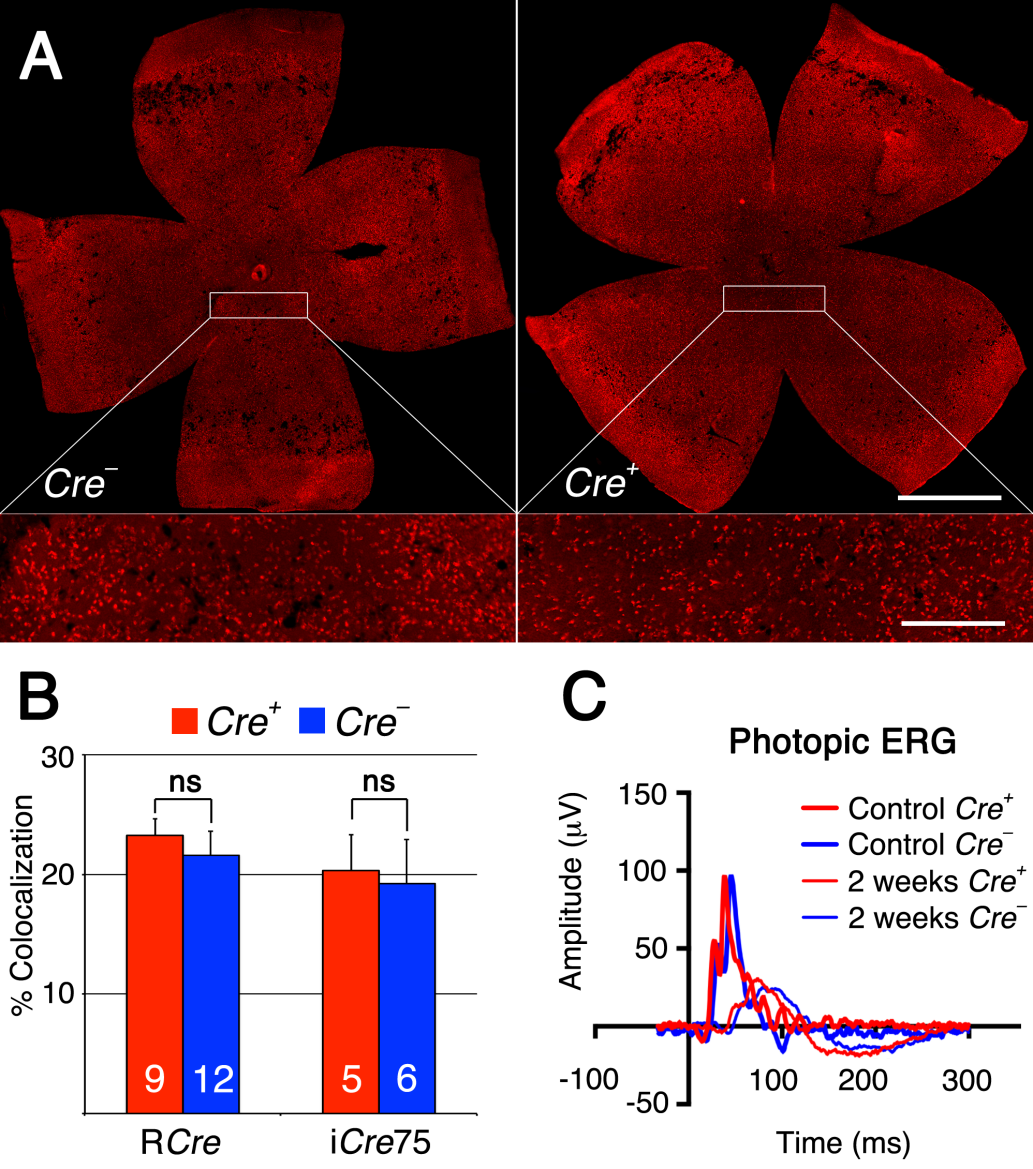
Rod survival does not promote cone survival **A.** Representative retinal flat mounts of R*Cre-* and R*Cre+* littermates harboring the *Tsc1fl/f* alleles showing no difference in the density of cones between R*Cre+* and R*Cre*- animals (see higher magnification in boxed area; red signal: cone arrestin). **B.** Percentage colocalization between cone arrestin and entire retinal surface area upon loss of *Tsc1* by the two rod-specific *Cre* lines indicated. **C.** Representative photopic ERG wave recordings prior (1 month of age) and 2 weeks post-injection of R*Cre-* and R*Cre+* littermates harboring the the *Tsc1fl/f* alleles. Diagram evaluating ONL thickness and radius of RPE damage for R*Cre* are shown in Figure 5A and 5B and for i*Cre*75 in Figure 6B and 6C respectively. (Number of animals analyzed is indicated in bar graph; ns: not significant).

To test if the same metabolic genes that were found to be upregulated in cones lacking *Tsc1* were also upregulated in rods after sodium iodate injection, we repeated the antibody stainings for HKII and G6PD. Because of the patchy expression of CRE recombinase in the R*Cre* line only that rod-specific *Cre*-driver line was used since CRE+ and CRE− cells are found within the same retina allowing for an internally controlled experiment. The antibody staining showed that both enzymes are upregulated in CRE+ areas (Figure 7) suggesting that the same mechanism that protects cones during degeneration also protect rods. Quantification of cone survival over the entire retinal surface area and the area corresponding to the area of RPE damage did not reveal any statistically significant difference between *Cre-* and *Cre+* animals for both rod-specific *Cre* driver lines (Figure 8; data not shown for RPE damage area). Overall the data show that promoting rod survival when RPE cells die does not promote cone survival. This suggests that once the RPE is gone photoreceptor survival is rather a cell autonomous process, and saving rods in AMD would have to occur prior to the loss of the RPE in order to save cones.

## DISCUSSION

The mouse model of acute sodium iodate induced RPE atrophy has principal similarities with GA, although the primary cause and time window of disease progression are quite different from those seen in humans. Areas devoid of RPE with open view of the choriocapillary bed, patchy loss of outer limiting membrane, and demise of PRs are manifestations of the human disease that were also observed by us in RPE and retinal flat mounts as well as retinal cross sections [10, 32-36]. However, while sodium iodate serves as a good model to study GA, variability in PR degeneration between different strains and individual animals must be accounted for when interpreting the data [37]. Therefore, only *Cre+* and *Cre-* littermates that suffered a similar extent of RPE damage were compared, in order to assure that changes in PR survival were due to the recombination of the conditional alleles rather than to differences in RPE damage. As our data shows the extent of RPE damage was always in agreement with the thickness of the ONL, except when rod death was delayed.

Loss of RPE cells in GA has been proposed to affect PR function and viability [15] because the RPE is involved in multiple processes that maintain photoreceptor homeostasis [38, 39]. To test if PRs could be rendered more resistant to the loss of RPE cells we increased the activity of mTORC1 in PRs, an approach that we have successfully employed to delay the death of nutrient deprived cones in retinitis pigmentosa [16, 17]. Because increased mTORC1 activity improves cone survival, while loss of mTORC1 accelerates cone death in the sodium iodate model but does not affect cone survival in wild-type mice [40], it is most likely that insufficient nutrient uptake by cones is the major contributor to cone loss once RPE cells die.

Rod PRs degenerate before cones in AMD [41, 42] and since rod loss always leads to cone loss [43], we tested if activating mTORC1 in rods would not only delay rod death but also benefit cone survival. Interestingly, while loss of *Tsc1* in rods did improve rod survival it did not alter cone survival, suggesting that rods themselves do not directly promote cone survival. Rather, PR survival is dependent on the RPE and once the RPE dies, both rods and cones suffer from a lack of adequate nutrient uptake. However, until the RPE dies cone survival is dependent on rod survival as the presence of rods helps maintaining cone outer segment - RPE interactions [17]. Therefore, increasing mTORC1 activity in PRs would be particularly beneficial for cones. In AMD, RPE cells do not die off abruptly as they do in the sodium iodate model, rather rods start to die before cones and RPE cells die [43]. Thus increased mTORC1 activity in PRs would directly promote rod and cone survival that is affected by a sick and underperforming RPE cell, and would indirectly promote cone survival as more rods remain present for a longer period of time [43].

In summary, increased cell-autonomous mTORC1 activity improves PR adaptability to the nutrient shortage caused by the loss of RPE cells, thereby promoting PR survival, while loss of mTORC1 results in a failure of PR to adapt demand with supply accelerating PR death. The findings negate a simple cone-rod dependency model and suggest that a lack of adequate nutrient supply is the driving force for PR death once RPE cells die. Furthermore, the data indicates that the two retinal degenerative diseases, AMD and retinitis pigmentosa, could be treated with the same therapeutic approach to extend vision [16], even though both diseases have different etiologies. Thus identifying either target genes or activators of mTORC1 that can promote PR survival may benefit individuals that suffer not only from inherited blinding diseases but also from age-related ones.

## MATERIALS AND METHODS

### Animals

All procedures involving animals were in compliance with the Association for Research in Vision and Ophthalmology (ARVO) Statement for the Use of Animals in Ophthalmic and Vision Research and were approved by the Institutional Animal Care and Use Committees (IACUC) of the University of Massachusetts Medical School. Animals were maintained on a 12-hour light/12-hour dark cycle with unrestricted access to food and water. Lighting conditions were kept constant in all cages, with illumination ranging between 10 and 15 lux. The *Ptenfl/fl*, *Tsc1fl/fl*, *Raptorfl/fl*, *Rictorfl/fl* mice and the cone-specific *Cre* line (here referred as M*Cre*) and rod-specific *Cre* lines (LMOP-Cre: here referred as R*Cre*; and i*Cre*75 here referred to as i*Cre*75) have all been described previously [25-27, 30, 31, 44]. Genotyping was performed as described in the original publications. In all instances only *Cre+* and *Cre-* male littermates were used for analysis. All mice were genotyped for absence of the *rd8* allele with mutation in the *Crb*1 gene [45] and none of the mice analyzed were albino.

### Sodium Iodate injections

Sodium iodate (catalog S4007; Sigma-Aldrich) was diluted in sterile phosphate-buffered saline (PBS) to a concentration 10 mg/mL. A single injection was administered to *Cre+* and *Cre*- littermates at 40 mg/kg via the tail vein in animals between 6-8 weeks of age with minimal time difference between *Cre+* and *Cre*- animals of the same conditional allele.

### Electroretinography (ERG)

Electroretinography recordings were performed as described earlier [40] using the Espion E3 console in conjunction with the ColorDome (Diagnosys LLC). The initial control ERG recordings were performed at least 24 hours prior to sodium iodate injections to allow animals to recover after anesthesia. Thereafter, ERGs were recorded from each experimental animal at 2 and 4 weeks post-injection. Photopic ERGs (cone response) were performed at a stimulus strength of 10 cd.s/m2, while scotopic ERGs (rod responses) were performed at a stimulus strength of 0.009 cd.s/m2.

### Tissue preparation, immunohistochemistry and fluorescent staining

Tissue samples with the R*Cre* and i*Cre*75 drivers were collected at 16 days (referred to 2 weeks in text) post-injection while all conditional alleles with the M*Cre* driver were collected at 30 ± 0.5 days (referred to 4 weeks in text) post-injection. Immunolabeling and fluorescent staining on retinal cryosections, retinal and RPE flat-mounts were performed as described previously [16, 17, 46] with the following modifications. One eye per animal was processed for cryosectioning the other was hemisected into neuroretinae and sclera-choroid-pigmented epithelium sample for flat mounts. The eye-cups for sectioning were fixed in cold 4% paraformaldehyde PBS buffered solution overnight, while neuroretinae and RPE to be flat mounted were fixed for 1 hour. After fixation, samples were washed in PBS and processed as described previously. Serial cryosections (12 μm) and flat mounts were mounted in Fluoro-Gel aqueous medium (catalog 17985-10, Electron Microscopy Science, Hatfield, PA). The following primary antibodies and dilutions were used: rabbit α-p-S6 (Ser240) (1:300; catalog 5364); rabbit α-HKII (1:300; catalog 2867) (both from Cell Signaling Technology); rabbit α-G6PD (1:300; catalog ab993; Abcam); rabbit α-GLUT1 (1:300; catalog GT11-A; Alpha Diagnostics); mouse α-*Cre* recombinase (1:500; catalog MMS-106P; Covance); rabbit α-cone arrestin (1:600; catalog AB15282; EMD Millipore); goat α-short wave length opsin (blue opsin) (1:200; catalog sc-14365; Santa Cruz); as well as rhodamine phalloidin (1:500; catalog R415; Life Technologies); and fluorescein-labeled peanut agglutinin lectin (PNA) (1:500; catalog FL-1071; Vector Laboratories). Nuclei were counterstained with 4′, 6-diamidino-2-phenylindole (DAPI) (catalog 9542; Sigma-Aldrich). All secondary antibodies (donkey) were purchased from Jackson ImmunoResearch and were purified F(ab)2 fragments that displayed minimal cross-reactivity with other species.

### Evaluation of RPE damage

The effectiveness of drug delivery through tail vein injection can vary quite a lot between individual animals and strains making it difficult to compare PR survival if the extend of RPE damage is not know. To minimize data variability and compare PR survival in retinas that suffered a similar extend of NaIO_3_-induced RPE damage, the neuroretina and the RPE of each eye-cup were carefully detached from each other and processed independently. The extent of RPE cell loss was first visualized by the absence of the glucose transporter 1 (GLUT1), which is normally expressed on the apical and basolateral RPE plasma membrane [23, 24], and by the absence of phalloidin labeled F-actin of RPE cell cytoskeleton delineating RPE cell boundary [47] (Figure 1). This allowed visualizing the integrity of the RPE layer and the underlying choriocapillary network. The central area of the RPE flat mount was characterized by extensive RPE atrophy with open view of the choriocapillary vascular bed (Figure 1). This area was followed by a narrow transition region with hypertrophied RPE cells, while the peripheral area had relatively intact RPE cells, where immunolabeling for GLUT1 was still clearly detectable (Figure 1). The radius of the surface area of NaIO_3_-induced RPE degeneration with open view of the underlying choroidal vasculature was measured, to calculate a RPE damage radius for each corresponding retina in order to determine which retinas to select for the PR survival analysis. The radius of RPE damage represents an average of 5 individual ratios, each obtained by measuring the radial extent of damage from the optic nerve head towards the *ora serrata* at a 72° interval divided by the full radial distance from the center of the optic nerve head to the *ora serrata* (Figure 1B). Therefore, the damage radius represents the radial length of the circular area around the optic nerve head that covers the RPE atrophy zone. A damage radius of 1 thus represents 100% RPE damage whereas a damage radius of 0 represents no damage. Only retinas for which the corresponding radius of RPE damage was 0.5 and greater were used for analysis. The highest radius of RPE damage calculated was 0.8, meaning that the surface area covered by 80% of the RPE radius was severely damaged. Finally, the average of the radii of RPE damage was calculated separately for all *Cre*- and all *Cre+* littermates, to ensure that in average a similar extent of RPE damage occurred between *Cre*- and *Cre+* littermates that were used to quantify PR survival.

### Quantification of cone and rod survival

Quantification of cone survival was performed as described previously [16, 17], with the following modification. The percentage of colocalization represents the percentage of the total retinal surface area that is covered by cones as seen by cone arrestin staining. Dormant cones or severely sick cones that stopped expressing cone arrestin are not detected by this method. The percentage colocalization calculated here is not reflective of the actual number of cones as in our previous publication [16] rather, it reflects the percentage of colocalization between the cone arrestin staining and the retinal surface area. Colocalization between the cone arrestin staining and the retinal surface area was calculated using CoLocalizer Pro software [48]. The reason for not converting the percentage colocalization into an actual number reflective of the percentage of surviving cones is that the cone arrestin staining changes across the retina following the extent of RPE damage, which differs from what is seen in the retinal degeneration 1 (*rd1*) mouse model of retinitis pigmentosa. In areas where the RPE is damaged cone arrestin staining is mainly in the cell body as the inner and outer segments are severely reduced in size. In peripheral areas, where no RPE damage is seen PR inner and outer segments look quite normal with most cone arrestin localized there. Thus it was not possible to properly calibrate the percentage of colocalization between cone arrestin staining and the retinal surface area with an actual number of surviving cones. Because the peripheral retina was better preserved due to the preservation of the peripheral RPE two colocalization values were calculated per retina: one value for the entire retinal surface area, and the other value for the retinal surface that corresponds to the area of RPE damage. Thus for each retina the corresponding RPE damage value was used to draw a circle on the retina and calculate the colocalization value within the circle (Figure 1). *Quantification of rod* survival was performed in two ways. In all cases measuring and averaging the thickness of the ONL from three adjacent radial cryo-sections of 12 μm thickness generated the ONL thickness diagram. All sections were through the head of the optic nerve in the nasal-temporal axis. Because the RPE damage value did not exceed 0.8, 18 measurements per retina spaced 200-μm apart were sufficient to cover the area of ONL damage. To calculate statistical significance between *Cre*+ and *Cre*- littermates averaged mean values of all 18 measurements were compared. In case of the R*Cre* driver line the percentage of CRE positive rods was calculated by counting manually across 3 radial sections of 12 μm thickness in injected and uninjected R*Cre+* animals all CRE positive cell and ONL nuclei. CRE positive cells were identified with α-*Cre* recombinase antibody while all nuclei were identified by DAPI stain. In the case of the i*Cre*75 all ONL nuclei were counted across one focal plain of one radial section per sample using IMARIS software. ONL nuclei were identified by DAPI.

### Imaging

All images were taken with a Leica DM5500 microscope. Retinal and pigmented epithelium-choriocapillary-sclera flat-mount images and sections for cell counting were acquired by tiling over the entire surface area with an automated scanning stage. All retinal flat-mounts are shown in the same orientation as indicated in Figure 2A.

### Statistical analysis

The two-tail unpaired Student's *t* Test was used for statistical analyses. *p*-values < 0.05 were considered statistically significant. Error bars represent SEM. Number of animals used in each experiment is indicated in the corresponding figure.

## SUPPLEMENTARY MATERIAL FIGURES AND TABLE


