# Supplementary material for: Improved cell metabolism prolongs photoreceptor survival upon retinal-pigmented epithelium loss in the sodium iodate induced model of geographic atrophy
Source: Oncotarget. 2016 Feb 11;7(9):9620–33. doi: 10.18632/oncotarget.7330 (PMC4891071; doi:10.18632/oncotarget.7330)
Supplement: Supplementary file 1 [file oncotarget-07-09620-s001.pdf]

Improved cell metabolism prolongs photoreceptor survival upon retinal-pigmented epithelium loss in the sodium iodate induced model of geographic atrophy

SUPPLEMENTAL MATERIAL

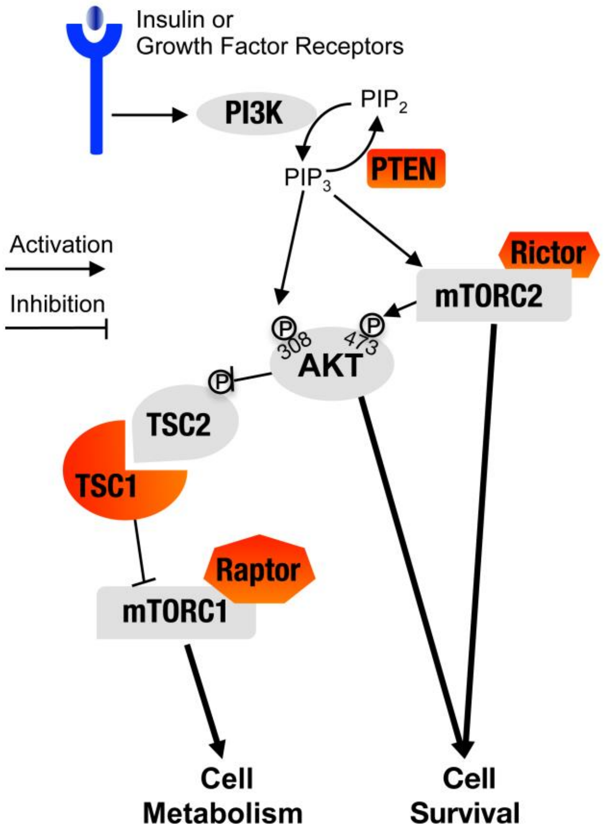

Supplemental Figure 1: Schematic of insulin/mTOR pathway. Upon insulin or growth factor binding to the receptor, phosphosinositide 3-kinase (PI3K) increases the concentration of the second messenger PIP_3. Increased PIP_3 levels promote mTORC2 and AKT activity. The phosphatase PTEN decreases PIP_3 levels thereby reducing mTORC2 and AKT activity and thus growth factor stimulation. Phosphorylation of AKT at Thr308 is sufficient to promote AKT activity towards the TSC2 protein tuberin, which once phosphorylated releases the inhibition of the TSC1-TSC2 complex on mTORC1 resulting in increased mTORC1 activity. Once AKT is phosphorylated on Thr308, its activity can further be increased by a subsequent phosphorylation

on Ser473 by mTORC2. Fully activated AKT promotes many pro-survival mechanisms. Similarly, activated mTORC2 can promote pro-survival mechanisms. In contrast activated mTORC1 controls mainly pro-growth processes. Genes deleted in study are shown in red.

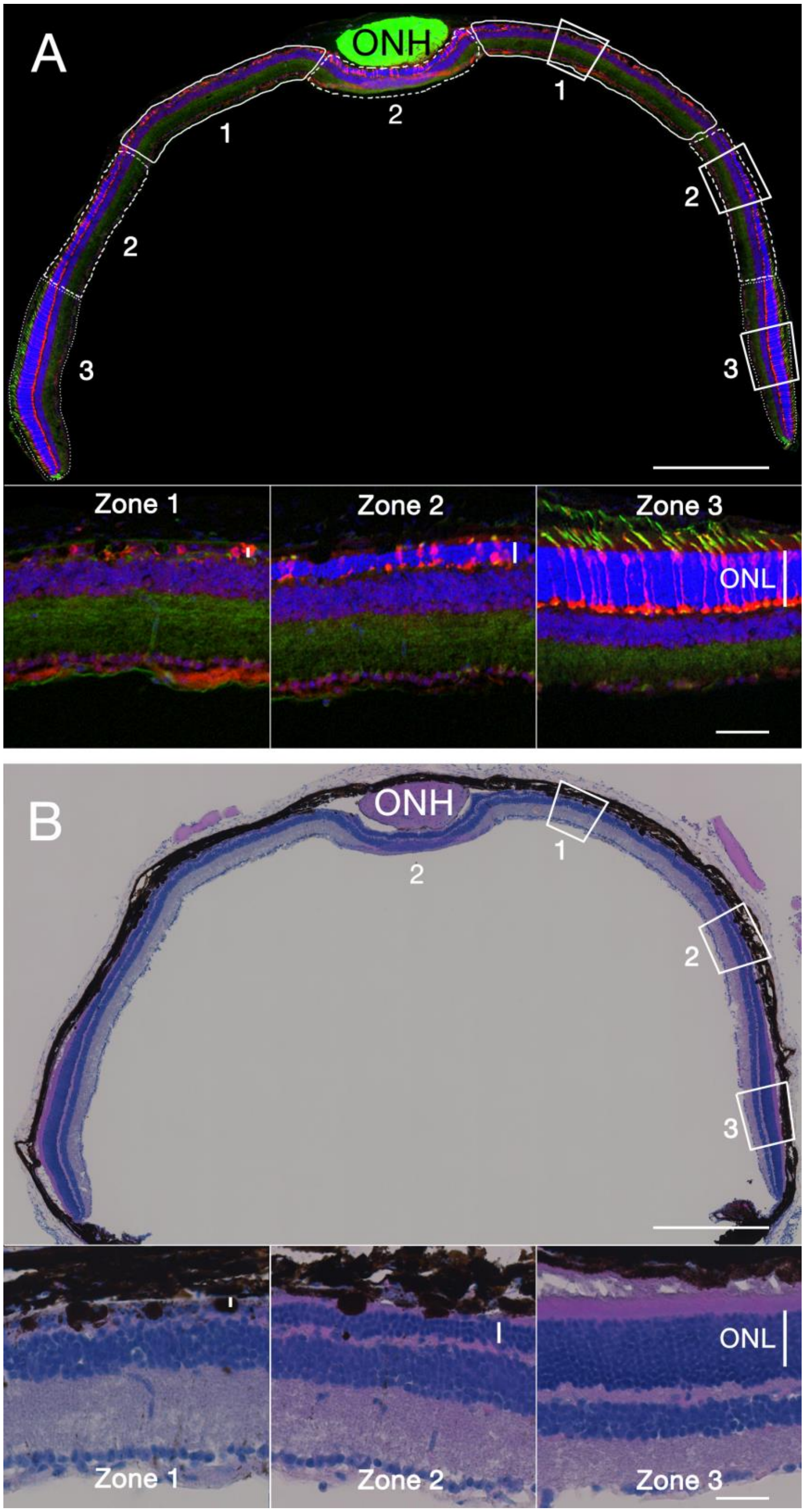

Supplemental Figure 2: Regional differences in photoreceptor survival across the neuroretina after sodium iodate injection. Retinal cross sections at 4 weeks post sodium iodate

injection showing the 3 different zones of degeneration. (**A, B**) Show an example from the *Tsc1*^{cKO} mouse retina in the nasal-temporal axis. (**A**) Shows immunofluorescence staining and (**B**) H&E staining of adjacent section. The RPE damage coefficient was 0.6. The area adjacent to the optic nerve head (zone 1) was characterized by only 1-3 rows of nuclei in the outer nuclear layer (ONL) with no outer segments and no apparent RPE present. In the following zone 2 the ONL was multiple rows thick with rare short distorted cone outer segments and hypertrophied GLUT1-negative RPE cells present, while the last zone appeared quite normal with minimal loss of PRs and an intact and presumably functional RPE. Interestingly, the area underneath the optic nerve head (ONH) was often less affected resembling the tissue in zone 2. Because the retinas were unaffected in the far periphery, ONL thickness was not recorded in the last 20% of the cross-sections. Interestingly, in the area encompassing the optic nerve head retinal morphology looked more similar to zone 2 than the adjacent zone 1. (**A**) Red signal: cone arrestin; green signal: PNA; blue signal: nuclear DAPI. Scale bars in overview image: 500 µm; scale bar in small panels 50 µm; vertical bars in small panels: thickness of ONL.

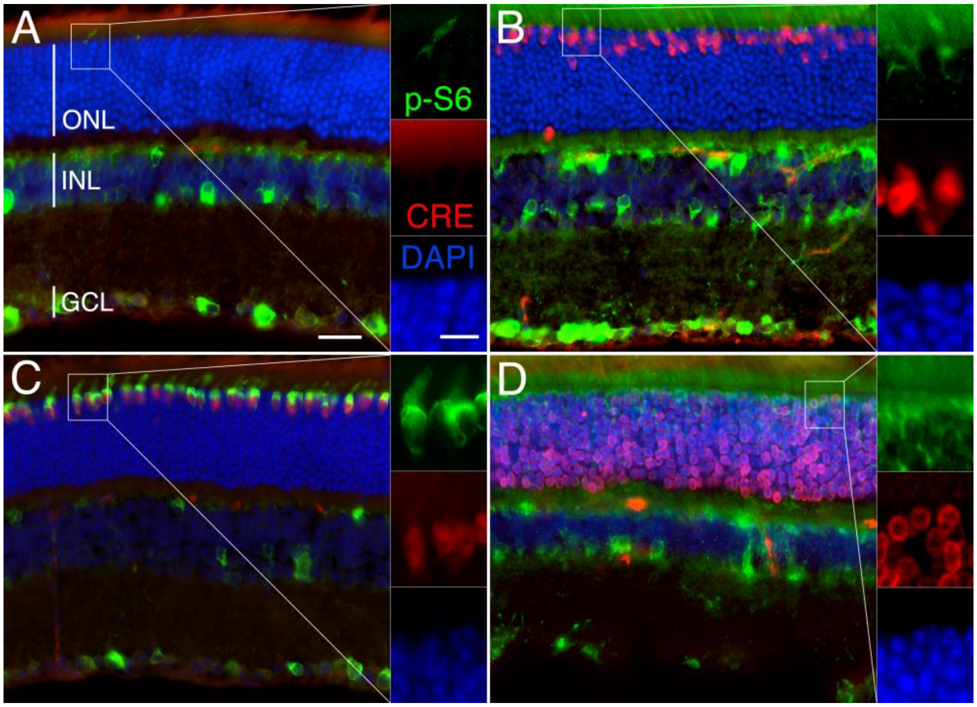

Supplemental Figure 3: Differential expression of p-S6 in cones upon loss of *Pten* and *Tsc1* and in rods upon loss of *Tsc1*. A-D. Retinal cross sections of uninjected mice at 2 months showing p-S6 in green, CRE in red and DAPI in blue. **A.** M*Cre*$^{-}$ control section of a *Tsc1*$^{fl/fl}$ animal showing that most of the p-S6 is located in the inner nuclear layer (INL) and ganglion cell layer (GCL). Rarely is some p-S6 seen in cones (see inset). **B.** Section of *Pten*^{cKO} showing weak p-S6 staining (green signal) in all cones (CRE: red signal). **C.** Section of *Tsc1*^{cKO} showing robust p-S6 staining (green signal) in all cones (CRE: red signal). **D.** Section of *Tsc1*$^{fl/fl}$ R*Cre*$^{+}$ mouse showing intermediate p-S6 staining (green signal) in CRE^{+} rods (red signal). Note that p-S6 signal in INL and GCL appears weaker in (**C**) due to a shorter exposure time because of the strong p-S6 staining in cones. p-S6 signal was the strongest after loss of *Tsc1* in cones (**C**) followed by loss of *Tsc1* in rods (**D**) and the loss of *Pten* in cones (**B**). Exposure time for p-S6 is the same in (**A,B,D**) and shorter in (**C**). Scale bars: 25 µm and 5 µm in inset. Vertical lines in (**A**) mark thickness of outer nuclear layer (ONL), inner nuclear layer (INL), ganglion cell layer (GCL).

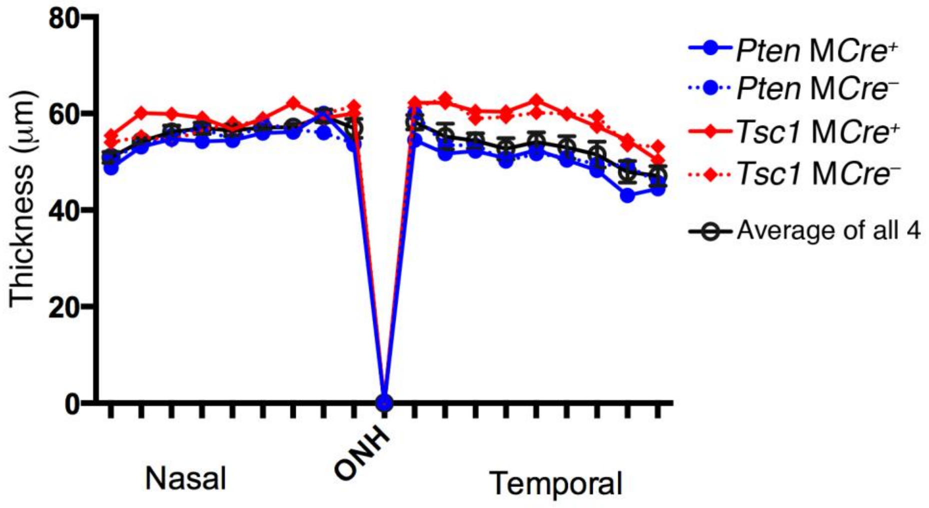

Supplemental Figure 4: Diagram of ONL thickness of *Pten*$^{fl/fl}$ and *Tsc1*$^{fl/fl}$ strains. Diagram showing thickness of ONL in uninjected M*Cre*$^{-}$ and M*Cre*$^{+}$ *Pten*$^{fl/fl}$ and *Tsc1*$^{fl/fl}$ animals indicating that in average (black line) the ONL thickness is comparable between these lines.

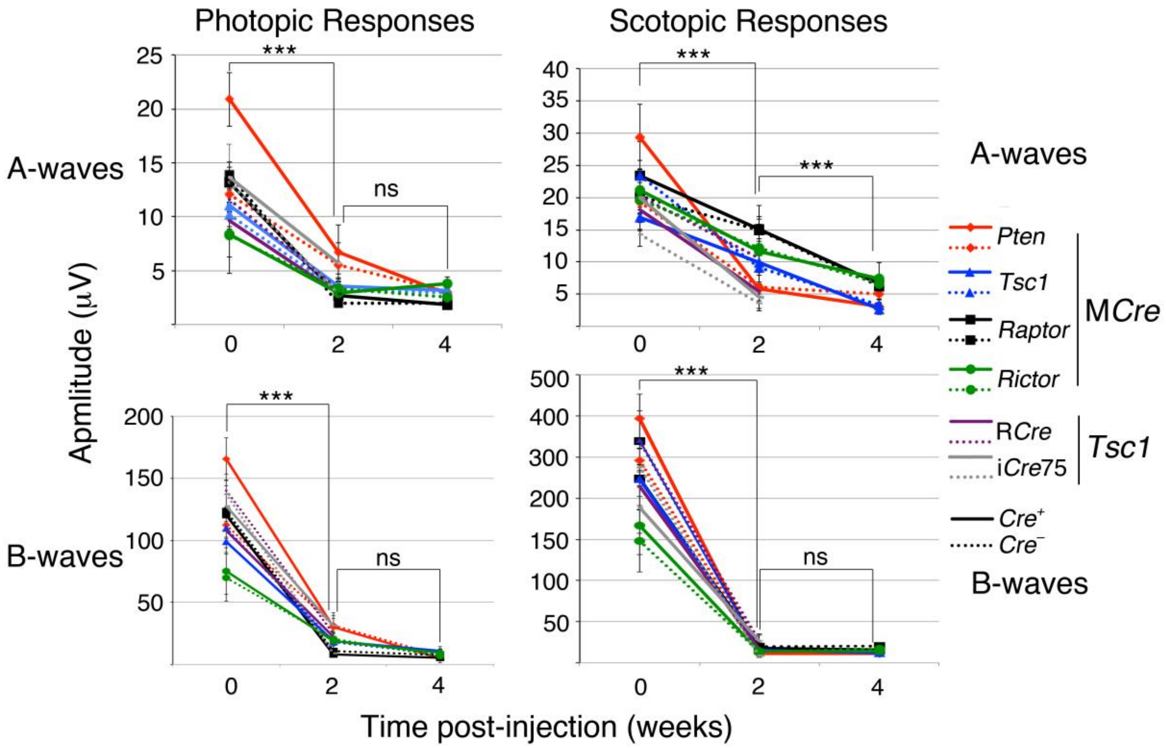

Supplemental Figure 5: Effect of sodium iodate on retinal function. Retinal function evaluated by ERG recordings prior to sodium iodate injection (1 months of age), 2 and 4 weeks post-injection. Retinal function declines rapidly after sodium iodate injection in all mouse strains studied. Both photopic cone responses and scotopic rod responses decline dramatically within the first two weeks even though the peripheral retina and RPE appear intact (Supplemental Figure 2). Responses of the intact peripheral retina can be seen in the scotopic a-wave where the decline in the rod response is rather linear suggesting that many functional photoreceptors are still present in the periphery at 2 weeks post sodium iodate injection. However, because sodium iodate also affects the connections between PR and bipolar cells, the b-wave in the scotopic response, which reflects bipolar cell activity, is already at its low point by 2 weeks post injection (Color combination for conditional alleles is indicated to the right; dotted line marks *Cre*$^{-}$ animals; ***: p<0.005).

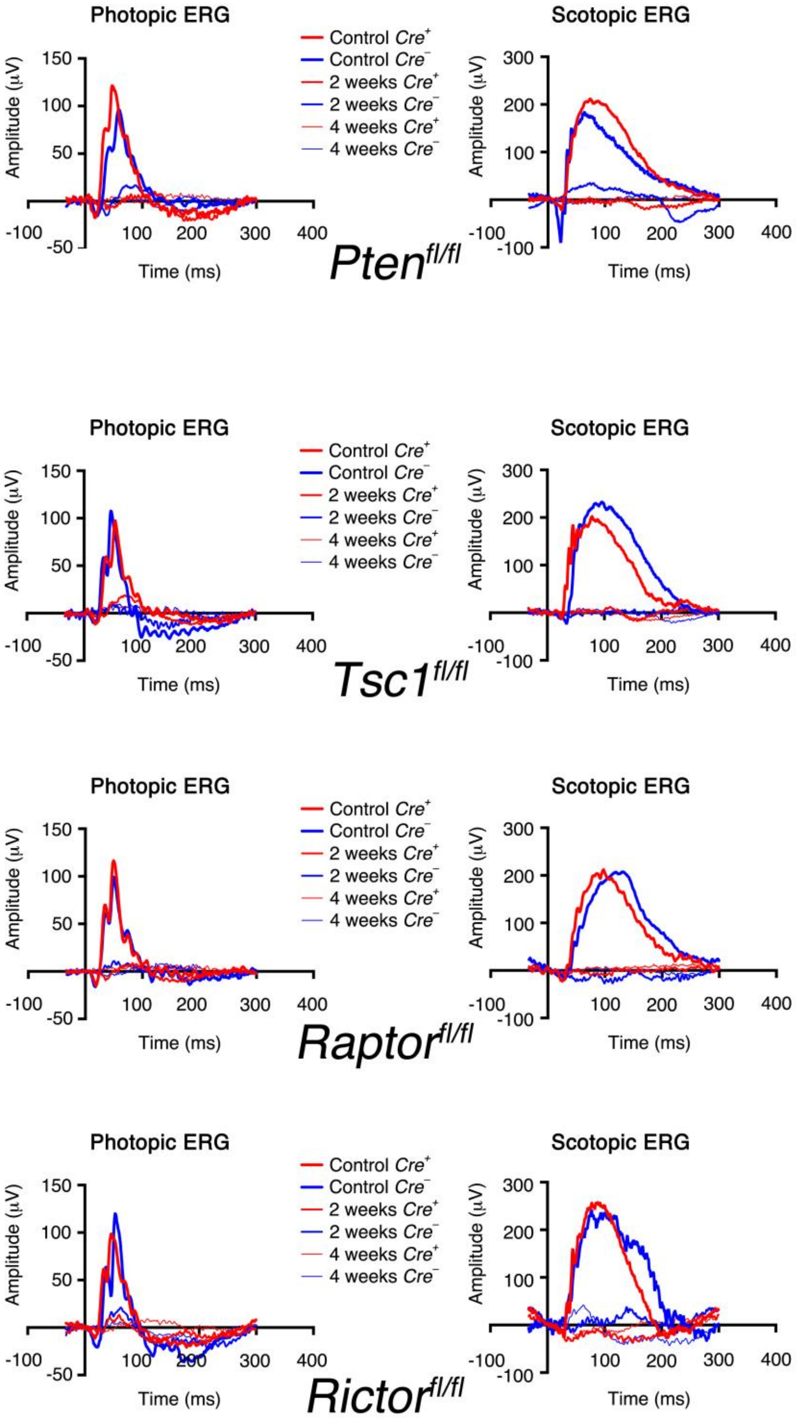

Supplemental Figure 6: Electroretinogram recordings. Representative ERG waves for scotopic and photopic ERGs showing dramatic decrease in a- and b-waves in all conditional backgrounds that were crossed to the cone specific M*Cre*. Conditional allele background is indicated in panel.

Supplemental Table 1: Percentage of mice successfully injected with sodium iodate having an RPE damage coefficient ≥ 0.5.

Strain	# of injected mice		# of failed injections		# of mice analyzed		Successful injections
	Cre^{+}	*Cre*$^{-}$	*Cre*$^{+}$	*Cre*$^{-}$	*Cre*$^{+}$	*Cre*$^{-}$	*Cre*$^{+}$ & *Cre*$^{-}$
Pten$^{fl/fl}$ M*Cre*	8	7	2	1	6	6	80%
Rictor$^{fl/fl}$ M*Cre*	7	5	1	0	6	5	92%
Raptor$^{fl/fl}$ M*Cre*	9	6	1	1	8	5	94%
Tsc1$^{fl/fl}$ M*Cre*	15	10	8	4	7	6	52%
Tsc1$^{fl/fl}$ R*Cre*	10	15	1	3	9	12	84%
Tsc1$^{fl/fl}$ i*Cre75*	7	7	2	1	5	6	79%
